# HLA-G 14 bp insertion/deletion polymorphism is not associated to proviral load levels and presence of HAM/TSP in Peruvian HTLV-1 infected individuals

**DOI:** 10.1186/1742-4690-8-S1-A122

**Published:** 2011-06-06

**Authors:** Sandra Morales, Jason Rosado, Giovanni López1, Jeroen Huyghe, Kristien Verdonck, Elsa González, Martin Tipismana, Daniel Clark, Guido Vanham, Eduardo Gotuzzo, Guy Van Camp, Lut Van laer, Michael Talledo

**Affiliations:** 1 Inst de Med Trop Alexander von Humboldt, Univ. Peruana Cayetano Heredia, Lima, Peru; 2 Dept of Med Genetics, Univ. of Antwerp, Antwerp, Belgium; 3 Virology Unit, Dept. of Microbiol., Inst. of Tropical Med., Antwerp, Belgium; 4 Fac de Ciencias y Filosofía Lab de Inves y Des, Univ. Peruana Cayetano Heredia, Lima, Peru; 5 Dept of Biomedical Sci., Univ. of Antwerp, Antwerp, Belgium

## Introduction

High HTLV-1 proviral load (PVL) has been associated with HAM/TSP disease in HTLV-1-infected individuals. Human leukocyte antigen (HLA) complex plays an important role in the immune response against virus-infected cells. One mechanism involves HLA-G binding to KIR2DL4 and modulates the activities of Natural Killer and CD8+ T cells. The 14-bp insertion/deletion in the 3’UTR exon 8 of HLA-G affects the HLA-G-mRNA-stability and therefore the HLA-G-protein levels. We analyzed the distribution of the 14-bp ins/del polymorphism among HTLV-1-infected individuals to evaluate its effect both on PVL and HAM/TSP.

## Subjects and methods

394 unrelated HTLV-1-infected Peruvian individuals were included in this analysis (254 asymptomatic carriers [AC] and, 140 HAM/TSP). HLA-G 14-bp ins/del and KIR2DL4 were genotyped with PCR specific primers. PVL was determined by real-time quantitative PCR using human the endogenous retrovirus 3 as reference gene. Associations of KIR-HLA-G ins/del with PVL or HAM/TSP were evaluated through multivariate logistic and linear regression analysis using R-software.

## Results and conclusions

KIR2DL4 was observed in all the individuals evaluated. The predominant genotype was +14-bp/-14-bp, both in AC (50.8%) and HAM/TSP (50.7%, P>0.05). HLA-G 14-bp ins/del showed no effect on PVL (P>0.05), nor with the presence of HAM/TSP in comparison to AC (P>0.05). This finding does not agree with the trend of high PVL observed for the -14-bp/-14-bp genotype, compared to +14-bp/-14-bp, +14-bp/+14-bp genotypes, in a Brazilian population. Our results stress the importance of replication studies in independent populations to demonstrate the association of host genetic factors with PVL or disease outcome in HTLV-I-infected subjects.

